# Autoimmune encephalitis in Latin America. Clinical features and outcomes in pediatric and adult populations: retrospective cohort of The REAL LABIC Project

**DOI:** 10.3389/fneur.2025.1647087

**Published:** 2025-09-02

**Authors:** Miguel A. Vences, Christoper A. Alarcon Ruiz, Mary Marcela Araujo Chumacero, Diego Canales-Pichen, Victor Saquisela, Jesús Domínguez-Rojas, Donoband Melgarejo, Saúl Reyes-Niño, Cintia Johnston, Maricela García-Arellano, Mercedes Amnely Suarez Loro, Cibele Lopes Queiroz de Lima, José Domingo Barrientos Guerra, Vanessa Cristina Waetge Pires de Godoy, William Bayona Pancorbo, Carla Gabriela Román Ojeda, Stefany Espinoza-Ramon, Karen Perales, Katya Granela, Jorge Flecha, Marlene Romero, Habib Georges Moutran Barroso, Jaime Toro, Werther Brunow Carvalho, Miguel Ángel Pelcastre Mejía, Kátia Yuri Xavier Mizumoto Soares, Ana Beatriz Sonta-Chan, Alejandra Gramajo-Juárez, Victor Edwin Ore Montalvo, Silvia Fabiola García Martínez, Belkis de la Candelaria, Daniel Agustin Godoy

**Affiliations:** ^1^Universidad Cesar Vallejo Piura, Piura, Peru; ^2^Neurociencia, Efectividad Clínica y Salud Pública, Universidad Científica del Sur, Lima, Peru; ^3^Hospital Nacional Edgardo Rebagliati Martins, Lima, Peru; ^4^Instituto Nacional de Salud del Nino, Lima, Peru; ^5^Hospital Central del Instituto de Prevision Social Dr Emilio Cubas, Asunción, Paraguay; ^6^Fundacion Santa Fe de Bogota, Bogotá, Colombia; ^7^Universidade de São Paulo, São Paulo, Brazil; ^8^Centenario Hospital Miguel Hidalgo, Aguascalientes, Mexico; ^9^Hospital Infantil Cândido Fontoura, São Paulo, Brazil; ^10^Hospital General San Juan de Dios, Guatemala City, Guatemala; ^11^Hospital Nacional Adolfo Guevara Velasco, Essalud, Cusco, Peru; ^12^Organización Clínica General del Norte de la Ciudad de Barranquilla Atlántico, Baranquilla, Colombia; ^13^Clinica General del Norte, Barranquilla, Colombia; ^14^Sanatório Pasteur, Catamarca, Argentina

**Keywords:** anti-N-methyl-D-aspartate receptor encephalitis, autoimmune diseases of the nervous system, encephalitis, critical care outcomes, Latin America

## Abstract

**Introduction:**

The Autoimmune Encephalitis Registry in Latin American countries (REAL LABIC Project) is an initiative created to conduct research focused on the epidemiological and clinical aspects of autoimmune encephalitis (AE) in the region. This study describes the sociodemographic profile, clinical presentation, treatment, and follow-up outcomes of patients diagnosed with AE across multiple reference centers from Latin America.

**Methods:**

A retrospective, multicenter cohort study was conducted in 10 hospitals across 6 countries from Latin America. Medical records of pediatric and adult patients hospitalized between July 2017 and June 2022 were reviewed. Inclusion criteria were diagnostic of probable or definite AE according to consensus diagnostic criteria by Graus et al. Comparative analyses were performed between pediatric and adult groups using hypothesis contrast tests.

**Results:**

The study included 165 patients, 57.6% were under 18 years of age. Confirmed AE was more frequent in pediatric patients, with anti-NMDA receptor antibodies identified in 53.5% of cases (CSF/serum). The median time from symptom onset to hospital admission was 8 days, significantly shorter in pediatrics (*p* = 0.027). A preceding viral prodrome was more common also in pediatrics (*p* = 0.003). ICU admission was required in 53.9% of cases, predominantly among pediatrics (*p* = 0.011). First-line immunotherapy was administered in 92.1% of patients, most commonly combining corticosteroids and intravenous immunoglobulin. Early initiation of treatment (≤7 days) was associated with better response in pediatrics. At six-month follow-up, 45.5% of patients showed persistent neurological disability (mRS: 2–5). Minor cognitive impairment was the most frequent long-term sequela. In-hospital complications were reported in 53.3% of cases, and the overall mortality rate was 19.4%.

**Conclusion:**

This is the first regional multicenter study of autoimmune encephalitis in Latin America highlighting the above findings. There were no significant differences in most of the analyzed variables between pediatric and adult populations. Future research should address the strengths and limitations of this registry with the aim of gaining a broader understanding of autoimmune encephalitis in our region.

## Introduction

Autoimmune encephalitis (AE) comprises a broad spectrum of disorders characterized by immune-mediated inflammation of the brain—with or without other regions of the central nervous system—leading to the subacute onset of neurological deficits (1). AE was not recognized as a distinct entity until 2007, when Dalmau et al. (2) isolated pathogenic neuronal surface antibodies. Prior to that, the etiopathogenic mechanism underlying these disorders were unknown.

The clinical manifestations of autoimmune encephalitis (AE) are highly heterogeneous, primarily characterized by the presence of cortical symptoms such as behavioral disturbances and epileptic seizures, which may be accompanied by subcortical signs including movement disorders or autonomic dysfunction. Due to its subacute course, clinical variability, and the range of possible presentations depending on the specific antibodies involved, diagnosis based on clinical presentation is challenging (1, 3).

Currently, many intracellular (Hu, Ma2, GAD) and surface (anti-NMDAR, AMPAR, LGI1, CASPR2, GABAR A, GABAR B, DPPX, glycine receptor, AQP4, MOG, GFAP, etc.) neuronal antibodies have been identified, with the most frequent being anti-NMDA receptor. Additionally, AE has been linked to the involvement of other systems, paraneoplastic processes, post-infectious conditions, or iatrogenic causes in the setting of exposure to immune-modulating agents (3).

Given the heterogeneity of clinical manifestations and antibodies associated with AE, in 2016, Graus et al. (4) established formal diagnostic criteria and levels of diagnosis certainty for this condition. The primary objective was to ensure early recognition and timely treatment, both of which are associated with better prognosis outcomes. The accurate clinical and epidemiological characterization of AE remains challenging, especially in low-resources settings. While the overall prevalence of AE is estimated at 13.7 per 100,000 (5), this is difficult to determine due to underreporting in low- and middle-income countries, such as those in the Latin American region with 0.16 per 100,000 person-years (6). This is thought to be due to various structural barriers hindering the proper diagnosis and treatment of AE (6, 7).

To address these gaps, the Autoimmune Encephalitis Registry in Latin American Countries (REAL LABIC Project) is an initiative of the Latin America Brain Injury Consortium (LABIC). This was established to conduct research focused on the epidemiological and clinical aspects of AE across centers from the region (8). The objective of this article is to describe these characteristics and therapeutic approaches, and to report the follow-up on clinical outcomes in pediatric and adult patients with AE.

## Materials and methods

### Study design and setting

We performed a retrospective, multicenter, observational cohort study to through the REAL LABIC Project to characterize the clinical features and outcomes of AE across Latin America during the period from July 2017 to June 2022. Ten tertiary hospitals with expertise in the clinical management and neurocritical care of patients with AE from Brazil, Colombia, Guatemala, Mexico, Paraguay, and Peru participated.

### Participants and sample selection

We included in this study all pediatric (under 18 years of age), and adult (18 years of age or older) patients diagnosed with possible AE (they were required to meet the following three criteria: 1. Subacute onset of working memory deficits, altered mental status or psychiatric symptoms. 2. At least one of the following: New focal CNS findings, recent-onset seizures, CSF pleocytosis, MRI features suggestive of encephalitis. 3. Exclusion of other causes), and then classified into probable or definite cases, according to consensus diagnostic criteria by Graus et al. (4), as recorded in medical records and the diagnosis was confirmed by the principal investigator of each center. All cases lacking neuronal antibody testing or with negative antibody results were subjected to review by the principal investigator of each participating center and by the research project’s central panel, comprised of specialists with clinical and research expertise in autoimmune encephalitis. Patients subsequently determined to have an alternative diagnosis other than AE, after medical record review, were excluded. Given the low incidence of AE, we employed a consecutive, discretionary and non-probabilistic sample selection to include every eligible AE case at participating centers during the study time.

### Data collection

The study was initially conducted in collaboration with coordinators from each of the participating countries of the LABIC consortium. The country coordinator identified possible participant hospitals from their home country. Then, a principal investigator from each hospital was invited to participate in the study. These investigators and their teams extracted de-identified patient data that met the study’s selection criteria using a standardized case report form. The principal investigators at each center were physicians with clinical expertise and training in autoimmune encephalitis. The study’s principal investigator conducted virtual training sessions for all participating investigators to collect data and record it in the database.

Collected study variables included:

- Sociodemographic characteristics (age, sex, simultaneous COVID-19 infection at symptom onset, and comorbidities).
- Clinical presentation (type of diagnosis, time in days from symptom onset to hospital admission and to diagnosis, viral prodrome, vital signs at admission, and clinical manifestations).
- Diagnostic support tests (neuronal antibody testing, electroencephalogram, and neuroimaging findings).
- Treatment aspects (time to treatment initiation, first-line acute treatment, maintenance therapy, use of antiviral or antibiotic therapy).
- Clinical evolution (ICU admission, need for mechanical ventilation, length of hospital stay, and use of antiseizure and antipsychotic medication).
- Clinical outcomes (In-hospital complications, clinical response rate according to the physician’s clinical assessment depending on the degree of improvement in the patient’s clinical manifestations, neurological disability with modified Rankin score, mortality, epilepsy control, needs for antiseizure and antipsychotic medication, neurocognitive disorders, and relapse) at discharge and up to 1 year of follow-up.

### Statistical analysis

All data were compiled into a single database for final analysis. Descriptive statistics were used to summarize patient characteristics, treatments, and outcomes. Categorical variables were summarized using frequencies and percentages, while quantitative variables were summarized using measures of central tendency and dispersion, including means and standard deviations or medians and interquartile ranges, depending on data distribution. Comparative analyses between pediatric and adult cohorts were performed using Chi-2 tests for categorical variables and Mann–Whitney U test for continuous variables, with a two-sided *α* of 0.05. All these previous analyses were conducted using Stata SE v18 software (StataCorp, Texas, the United States) Tables and graphs were created to summarize the key findings on Microsoft Excel (Microsoft Corporation, Washington, the United States). Graphs compared treatment response among cohorts and treatment scheme at each follow-up.

### Ethical considerations

Informed consent was waived given the retrospective nature of the study. The confidentiality of patient data was strictly maintained, with anonymization through alphanumeric coding, accessible only to the principal investigators at each participating hospital.

The study adhered to local regulations of each participating center. The Autoimmune Encephalitis Registry in Latin American countries (REAL LABIC Project) was registered in the research project repository from the Universidad Cesar Vallejo and was approved by its Institutional Review Board (050-CEI-EPM-UCV-2023). Then, before implementation, the project was approved by the Institutional Review Boards of each participating hospital.

## Results

A total of 165 participants were included in this multicenter registry, of whom 95 (57.6%) were pediatric cases (<18 years). Nearly half of the cohort were recruited from Peruvian institutions (*n* = 81, 49.1%). In the pediatric subgroup, the majority were male (53.7%), whereas females were more prevalent in the adult participants (57.1%). The most frequent comorbidities included hypertension (observed only in adults), COVID-19 infection (6.7%), obesity (6.7%), and other autoimmune diseases (6.7%) (Table 1).

**Table 1:** Sociodemographic characteristics of patients with autoimmune encephalitis (*n* = 165).

Characteristic	Total, *n* (%)	Pediatrics, *n* (%)	Adults, *n* (%)
Country			
Peru	81 (49.1)	48 (59.3)	33 (40.7)
Brazil	23 (13.9)	23 (100.0)	0 (0.0)
Colombia	23 (13.9)	5 (21.7)	18 (78.3)
Mexico	17 (10.3)	15 (88.2)	2 (11.8)
Guatemala	13 (7.9)	4 (30.8)	9 (69.2)
Paraguay	8 (4.9)	0 (0.0)	8 (100.0)
Sex			
Female	84 (50.9)	44 (52.4)	40 (47.6)
Male	81 (49.1)	51 (63.0)	30 (37.0)
Age (years)^a^	16 (8–34)	10 (5–14)	38 (27–62)
COVID-19 vaccination	35 (21.2)	19 (54.3)	16 (45.7)
Hypertension	14 (8.5)	0 (0.0)	14 (100.0)
COVID-19 infection	11 (6.7)	5 (45.5)	6 (54.6)
Obesity	11 (6.7)	4 (36.4)	7 (63.6)
Other autoimmune disease	11 (6.7)	8 (72.7)	3 (27.3)
Dyslipidemia	4 (2.4)	1 (25.0)	3 (75.0)
Lung disease	4 (2.4)	4 (100.0)	0 (0.0)
Heart failure	1 (0.6)	0 (0.0)	1 (100.0)

a Median and interquartile range (IQR).

Of the 165 registered cases, nearly half of them met criteria for definite AE (*n* = 78, 47.3%), with a higher proportion in the pediatric cohort. The predominant presentation was encephalitis, particularly in the adult cohort. While the pediatric group exhibited combined syndromes with cerebellar, striatal, or diencephalic. The median time from symptom onset to hospital admission was 8 days, but significantly shorter in pediatrics (median 7 days) than adults (median 14 days) (*p* = 0.027). A viral prodrome before symptom onset was more frequent in the pediatric group (*p* = 0.003), as were fever at admission, altered level of consciousness, akinetic mutism, movement disorders, hyperreflexia, motor and sensory deficits, cranial nerve palsies, and ataxia (Table 2).

**Table 2:** Clinical characteristics of patients with autoimmune encephalitis (*n* = 165).

Clinical features	Total, *n* (%)	Pediatrics, *n* (%)	Adults, *n* (%)	*p* value
Type of diagnosis				0.108
Probable	87 (52.7)	45 (47.4)	42 (60.0)	
Definite	78 (47.3)	50 (52.6)	28 (40.0)	
Clinical presentation (*n* = 124)				**0.002**
Limbic encephalitis	42 (33.9)	22 (29.0)	20 (41.7)	
Cortical/subcortical encephalitis	41 (33.1)	18 (23.7)	23 (47.9)	
Combined presentation	31 (25.0)	28 (36.8)	3 (6.25)	
Cerebellar	5 (4.0)	4 (5.3)	1 (2.1)	
Striatal	2 (1.6)	1 (1.3)	1 (2.1)	
Meningoencephalitis	2 (1.6)	2 (2.6)	0 (0.0)	
Diencephalic	1 (0.8)	1 (1.3)	0 (0.0)	
Time to admission (days)^a^	8 (4–18)	7 (4–15)	14 (5–21)	**0.027**
Time to diagnosis (days)^a^	13 (7–25)	10 (6–24)	16 (10–28)	0.066
Viral prodrome	40 (24.2)	31 (32.6)	9 (12.9)	**0.003**
SBP >140 mmHg at admission (*n* = 155)	12 (7.7)	4 (4.6)	8 (11.9)	0.088
DBP >90 mmHg at admission (*n* = 143)	9 (6.3)	4 (5.1)	5 (7.7)	0.530
Fever	48 (29.1)	42 (44.2)	6 (8.6)	**<0.001**
Seizures	123 (74.6)	75 (79.0)	48 (68.6)	0.130
Behavioral disorder	121 (73.3)	70 (73.7)	51 (72.9)	0.905
Cognitive impairment	91 (55.2)	48 (50.5)	43 (61.4)	0.164
Altered level of consciousness	90 (54.6)	63 (66.3)	27 (38.6)	**<0.001**
Movement disorder	77 (46.7)	53 (55.8)	24 (34.3)	**0.006**
Headache	65 (39.4)	36 (37.9)	29 (41.4)	0.646
Sleep disorder	63 (38.2)	42 (44.2)	21 (30.0)	0.063
Hyperreflexia	50 (30.3)	36 (37.9)	14 (20.0)	**0.013**
Motor deficit	49 (29.7)	36 (37.9)	13 (18.6)	**0.007**
Ataxia	43 (26.1)	32 (33.7)	11 (15.7)	**0.009**
Visual hallucinations	41 (24.9)	26 (27.4)	15 (21.4)	0.383
Dysautonomia	40 (24.2)	27 (28.4)	13 (18.6)	0.145
Aphasia	40 (24.2)	28 (29.5)	12 (17.1)	0.068
Dysarthria	32 (19.4)	23 (24.2)	9 (12.9)	0.068
Akinetic mutism	28 (17.0)	24 (25.3)	4 (5.7)	**0.001**
Babinski sign	27 (16.4)	19 (20.0)	8 (11.4)	0.141
Auditory hallucinations	27 (16.4)	16 (16.8)	11 (15.7)	0.847
Prefrontal syndrome	27 (16.4)	19 (20.0)	8 (11.4)	0.141
Cranial nerve palsies	24 (14.6)	20 (21.1)	4 (5.7)	**0.006**
Sensory deficit	22 (13.3)	20 (21.1)	2 (2.9)	**0.001**
Meningism	21 (12.7)	10 (10.5)	11 (15.7)	0.323
Dysmetria	13 (7.9)	10 (10.5)	3 (4.3)	0.141

a Median and interquartile range.

*p*-value according to the Chi-2 hypothesis test or Mann–Whitney U test; SBP, Systolic blood pressure; DBP, Diastolic blood pressure.Bold values indicates statistical significance *p* ≤ 0.05.

Neural antibody testing was performed in two thirds of the patients (the samples used were CSF or serum and the testing methods were heterogeneous, depending on the laboratory that processed the sample), with anti-NMDA receptor antibodies detected most frequently (53.5% of tested cases). Three patients were positive to LGI-1 antibody and Anti-GAD 65/67, each. Then, Anti-Yo, CASPR2, and GABA-B were positive in one patient, each. Electroencephalography (most of at least 60 min) was obtained in 84.8% of patients, brain computed tomography in 86.1%, and brain magnetic resonance imaging (MRI 1.5/3T) in 77.0%. Normal MRI findings were significantly more frequent in pediatrics, whereas adults more often demonstrated abnormalities (*p* = 0.023) (Table 3).

**Table 3:** Complementary test and treatments of patients with autoimmune encephalitis (*n* = 165).

Laboratory test and treatment	Total, *n* (%)	Pediatrics, *n* (%)	Adults, *n* (%)	*p* value
Neural antibodies				0.058
Not performed	64 (38.8)	35 (36.8)	29 (41.4)	
Negative	37 (22.4)	23 (24.2)	14 (20.0)	
Anti-NMDA receptor	54 (32.7)	35 (36.8)	19 (27.1)	
Another antibody^a^	10 (6.1)	2 (2.1)	8 (11.4)	
Electroencephalogram				
Not performed	25 (15.2)	14 (14.7)	11 (15.7)	0.229
Normal	37 (22.4)	17 (17.9)	20 (28.6)	
Pathological	103 (62.4)	64 (67.4)	39 (55.7)	
Brain CT				0.351
Not performed	23 (13.9)	15 (15.8)	8 (11.4)	
Normal	112 (67.9)	66 (69.5)	46 (65.7)	
Pathological	30 (18.2)	14 (14.7)	16 (22.9)	
Brain MRI				**0.023**
Not performed	38 (23.0)	27 (28.4)	11 (15.7)	
Normal	74 (44.9)	45 (47.4)	29 (41.4)	
Pathological	53 (32.1)	23 (24.2)	30 (42.9)	
Brain PET scan				0.118
Not performed	149 (90.3)	88 (92.6)	61 (87.1)	
Normal	6 (3.6)	1 (1.1)	5 (7.1)	
Pathological	10 (6.1)	6 (6.3)	4 (5.7)	
Time to treatment, days (*n* = 130)^b^	12 (7–24)	10 (5–17)	17 (10–33)	**<0.001**
Type of acute treatment (*n* = 160)				
Corticosteroid pulse therapy	133 (80.6)	83 (87.4)	50 (71.4)	**0.010**
Intravenous immunoglobulin	128 (77.6)	72 (75.8)	56 (80.0)	0.522
Plasma exchange	34 (20.6)	20 (21.1)	14 (20.0)	0.869
Antibiotic	52 (31.5)	41 (43.2)	11 (15.7)	**<0.001**
Acyclovir	67 (40.6)	50 (52.6)	17 (24.3)	**<0.001**
Type of maintenance treatment (*n* = 48)				
Rituximab	36 (21.8)	22 (23.2)	14 (20.0)	0.627
Cyclophosphamide	18 (10.9)	6 (6.3)	12 (17.1)	**0.027**
Mycophenolate mofetil	4 (2.4)	2 (2.1)	2 (2.9)	1.000
Azathioprine	1 (0.6)	0 (0.0)	1 (1.4)	0.312
ICU admission	92 (55.8)	61 (64.2)	31 (44.3)	**0.011**
Need for mechanical ventilation	64 (38.8)	40 (42.1)	24 (34.3)	0.308
Length of hospitalization (*n* = 145)^b^	30 (16–49)	30 (16–45)	32 (16–49)	0.445
Use of antiseizure medication	104 (63.0)	61 (64.2)	43 (61.4)	0.714
Use of antipsychotic medication	91 (55.1)	54 (56.8)	37 (52.9)	0.611

*p*-value according to the Chi-square test or Mann–Whitney U test. CT, Computed axial tomography; ICU, Intensive Care Unit; MRI, Magnetic resonance imaging; NMDA receptor, N-methyl-D-aspartate receptor; PET scan, Positron Emission Tomography scan.

a There is one subject positive to anti-NMDA receptor and to anti-GAD 65/67.

b Median and interquartile range. Bold values indicates statistical significance *p* ≤ 0.05.

A total of 160 patients (97.0%) received acute-phase treatment, including immunotherapy and/or anti-infective agents. The most commons immunotherapies were corticosteroid pulse therapy (80.6%) and intravenous immunoglobulin (IVIG) (77.6%). Additionally, 31.5% received empirical antibiotics and 40.6% were treated with acyclovir—interventions that were predominantly administered in the pediatric cohort. Following the acute phase, 29.1% of patients were started on maintenance immunotherapy, with rituximab and cyclophosphamide being the most commonly used agents. Overall, more than half of the patients required admission to the intensive united care, a requirement significantly more common among pediatric cases (*p* = 0.011) (Table 3).

In the acute phase, first line treatment immunotherapy consisted of monotherapy (IVIG, corticosteroid pulse therapy, or plasma exchange) or either in combination. The criteria for this decision were heterogeneous among the participating centers and were according to their internal protocols and individual case severity. Overall, 152 patients (92.1%) received first-line immunotherapy. Of these, 91 (59.9%) received dual therapy with corticosteroid pulses and IVIG (59.6% of children vs. 60.3% of adults), 20 (13.2%) received triple therapy with corticosteroid pulses, IVIG, and plasma exchange (14.6% of children vs. 11.1% of adults), 14 (9.2%) received IVIG alone (5.6% of children vs. 14.3% of adults), and 13 (8.6%) received corticosteroid pulses alone (12.4% of children vs. 3.2% of adults). here were no significant differences in treatment regimens between the pediatric and adult cohorts (*p* = 0.093).

**Figure 1:**
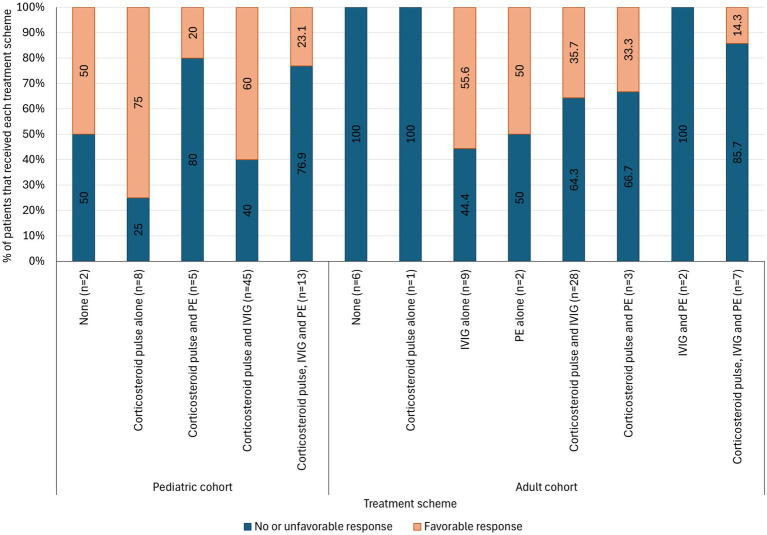
Clinical response rates by treatment scheme in pediatrics and adults with autoimmune encephalitis (*n* = 131). IVIG, Intravenous immunoglobulin; PE, Plasma exchange.

Data of first line treatment immunotherapy scheme and their clinical response rate were available for 131 patients (79.4%). In the pediatric cohort, favorable responses were observed in 75% (6/8) of those receiving corticosteroid pulse monotherapy, 60.0% (27/45) of those receiving combined corticosteroid pulses and IVIG, and 23.1% (3/13) of those receiving triple therapy. Among adults, favorable responses were recorded in 55.6% (5/9) of IVIG monotherapy recipients, 35.7% (10/28) of those treated with dual corticosteroid pulses plus IVIG, and 14.3% (1/7) of those receiving triple therapy (Figure 1).

**Figure 2:**
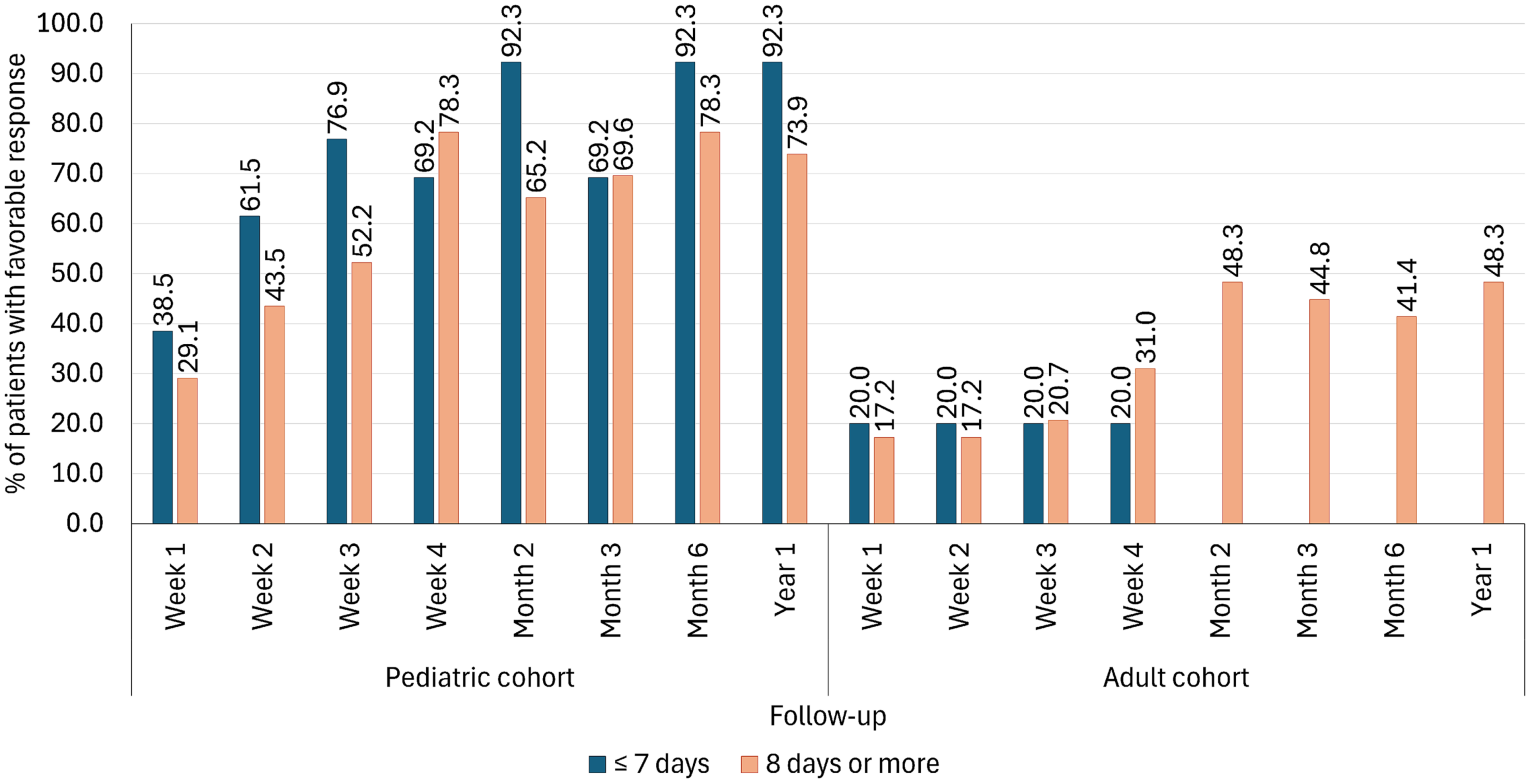
Favorable response rates during one-year follow-up based on treatment initiation timing in pediatrics and adults with autoimmune encephalitis (*n* = 70).

The time from symptom onset to treatment initiation was documented in 78.8% of cases (76.9% of pediatrics and 81.4% of adults). The median time was significantly shorter in the pediatric cohort than in adults (10 days in pediatrics and 17 days in adults). Long-term follow-up, ranging from 1 week to 1 year, was available in 42.4% of patients (36 pediatrics and 34 adults). In pediatric patients, those who began treatment within 7 days of symptom onset demonstrated higher rates of favorable response than those treated after 8 days. This advantage persisted through 1 year of follow up. By contrast, in adults, the timing of treatment initiation did not significantly influence the frequency of favorable response (Figure 2).

**Figure 3:**
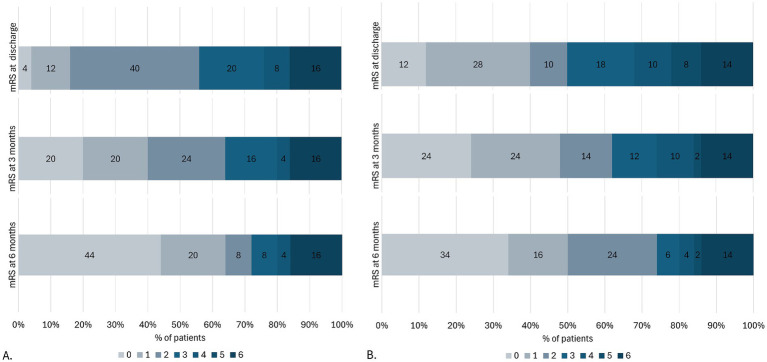
Neurological disability status at discharge and follow-up in pediatric **(A)** and adult patients **(B)** with autoimmune encephalitis (*n* = 75). mRS, Modified Rankin scale.

Neurological disability (mRS: 2–5) within 6 months of follow-up was reported in 45.5% of cases (25 pediatrics and 50 adults). Both pediatrics and adults showed progressive improvement in the degree of disability at each follow-up interval. By 6 months, 64% of pediatric patients had a modified Rankin Scale score of 0 or 1, indicating minimal or no disability, compared with 50% of adults. No additional deaths were reported after hospital discharge (Figure 3).

More than half of the patients (53.5%) experienced at least one in-hospital complication. Most common neurological complications included status epilepticus (30 in children cohort and 24 in adult cohort), intracranial hypertension (2 in children cohort and 3 in adult cohort), and ischemic stroke (3 in children cohort and 1 in adult cohort). Most common infectious complications included pneumonia (21 in children cohort and 28 in adult cohort) and urinary tract infection (4 in children cohort and 3 in adult cohort). In-hospital mortality occurred in 19.4% of patients (Table 4). During the six-month follow-up, one-third of the patients had seizure recurrence, and over half continued to require antiseizure and antipsychotic medications. At one-year follow-up, seizure recurrence had decreased to 14.3%, although half remained on long-term medications. Persistent cognitive deficits were documented in several cases, and clinical relapse occurred in 13% within the first year. There were no significant differences in these outcomes between pediatric and adult cohorts (Table 4). Additionally, we did not find association between relapse within the first year with sex (*p* = 0.770), COVID-19 vaccination (*p* = 0.550), probable or confirmed diagnosis (*p* = 0.489), or maintenance treatment (0.966).

**Table 4:** Clinical outcomes of patients with autoimmune encephalitis (*n* = 165).

Clinical outcomes	Total, *n* (%)	Pediatrics, *n* (%)	Adults, *n* (%)	*p* value
In-hospital complication	88 (53.3)	51 (53.7)	37 (52.9)	0.916
Neurological complication	67 (40.6)	40 (42.1)	27 (38.6)	0.648
Infectious complication	64 (38.8)	32 (33.7)	32 (45.7)	0.117
Complication of immobility (pressure ulcers)	32 (19.4)	20 (21.1)	12 (17.1)	0.530
In-hospital death	16 (9.7)	9 (9.5)	7 (10.0)	0.910
Seizure recurrence at 6 months (*n* = 116)	39 (33.6)	24 (32.4)	15 (35.7)	0.719
Seizure recurrence at 1 year (*n* = 105)	15 (14.3)	10 (15.4)	5 (12.5)	0.682
Need for antiseizure medication at 6 months (*n* = 90)	57 (63.3)	27 (58.7)	30 (68.2)	0.351
Need for antiseizure medication at 1 year (*n* = 80)	41 (51.3)	17 (41.5)	24 (61.5)	0.073
Need for psychotropic medication at 6 months (*n* = 100)	57 (57.0)	33 (56.9)	24 (57.1)	0.980
Need for psychotropic medication at 1 year (*n* = 92)	43 (46.7)	25 (47.2)	18 (46.2)	0.923
Cognitive impairment at 1 year (*n* = 85)				0.140
Minor	49 (57.6)	27 (60.0)	22 (55.0)	
Major	9 (10.6)	2 (4.4)	7 (17.5)	
Relapse at 1 year (*n* = 123)	16 (13.0)	9 (12.9)	7 (13.2)	0.954

*p*-value according to the Chi-2 hypothesis test. Bold values indicates statistical significance *p* ≤ 0.05.

In the pediatric cohort, six patients (6.3%) received no immunotherapy; 16 (16.8%) received monotherapy (corticosteroid pulses, *n* = 11; IVIG, *n* = 5); 60 (63.2%) received dual therapy—corticosteroid pulses + IVIG (*n* = 53), corticosteroid pulses + plasma exchange (*n* = 6), or IVIG + plasma exchange (*n* = 1); and 13 (13.7%) received triple therapy. Patients treated with dual or triple therapy had a significantly lower in-hospital mortality rate than those receiving monotherapy. In the adult cohort, seven patients (10.0%) received no immunotherapy; 13 (18.6%) received monotherapy (IVIG, n = 9; corticosteroid pulses, *n* = 2; plasma exchange, *n* = 2); 43 (61.4%) received dual therapy—corticosteroid pulses + IVIG (*n* = 38), corticosteroid pulses + plasma exchange (*n* = 3), or IVIG + plasma exchange (*n* = 2); and seven (10.0%) received triple therapy. In this group, dual or triple therapy was associated with a significantly lower rate of cognitive impairment at 1 year compared to monotherapy (Table 5).

**Table 5:** Clinical outcomes of patients with autoimmune encephalitis according to treatment scheme (*n* = 165).

Clinical outcomes	None, *n* (%)	Monotherapy, *n* (%)	Dual therapy, *n* (%)	Triple therapy, *n* (%)	*p*-value
Children cohort (*n* = 95)					
In-hospital complication	3 (50.0)	9 (56.3)	29 (48.3)	10 (76.9)	0.309
In-hospital death	3 (50.0)	3 (18.8)	3 (5.0)	0 (0.0)	**0.001**
Seizure recurrence at 6 months (*n* = 74)	2 (66.7)	4 (36.4)	15 (31.3)	3 (25.0)	0.569
Seizure recurrence at 1 year (*n* = 65)	1 (33.3)	2 (25.0)	7 (15.6)	0 (0.0)	0.400
Need for antiseizure medication at 6 months (*n* = 46)	2 (100.0)	3 (100.0)	19 (50.0)	3 (100.0)	0.078
Need for antiseizure medication at 1 year (*n* = 41)	2 (100.0)	2 (100.0)	12 (33.3)	1 (100.0)	0.045
Need for psychotropic medication at 6 months (*n* = 58)	2 (100.0)	2 (50.0)	20 (48.8)	9 (81.8)	0.140
Need for psychotropic medication at 1 year (*n* = 53)	1 (50.0)	2 (50.0)	13 (34.2)	9 (100.0)	**0.005**
Any cognitive impairment at 1 year (*n* = 45)	1 (100.0)	2 (50.0)	18 (58.1)	8 (88.9)	0.282
Relapse at 1 year (*n* = 70)	1 (33.3)	3 (30.0)	5 (11.1)	0 (0.0)	0.131
Adult cohort (*n* = 70)					
In-hospital complication	4 (57.1)	9 (69.2)	19 (44.2)	5 (71.4)	0.294
In-hospital death	1 (14.3)	0 (0.0)	5 (11.6)	1 (14.3)	0.603
Seizure recurrence at 6 months (*n* = 42)	0 (0.0)	3 (33.3)	11 (40.7)	1 (33.3)	0.574
Seizure recurrence at 1 year (*n* = 40)	1 (20.0)	0 (0.0)	4 (16.0)	0 (0.0)	0.580
Need for antiseizure medication at 6 months (*n* = 44)	1 (25.0)	7 (77.8)	19 (67.9)	3 (100.0)	0.156
Need for antiseizure medication at 1 year (*n* = 39)	1 (25.0)	5 (71.4)	16 (64.0)	2 (66.7)	0.450
Need for psychotropic medication at 6 months (*n* = 42)	1 (25.0)	4 (50.0)	17 (63.0)	2 (66.7)	0.505
Need for psychotropic medication at 1 year (*n* = 39)	0 (0.0)	3 (42.9)	14 (56.0)	1 (33.3)	0.201
Any cognitive impairment at 1 year (*n* = 40)	0 (0.0)	6 (100.0)	22 (78.6)	1 (50.0)	**0.003**
Relapse at 1 year (*n* = 53)	0 (0.0)	0 (0.0)	7 (21.2)	0 (0.0)	0.180

*p*-value according to the Chi-2 hypothesis test.

## Discussion

### Key findings

In this first regional and multicenter registry of AE in Latin America, we characterized 165 pediatric and adult cases across 10 centers from six different countries in the region. Nearly half of the cases were confirmed cases according to consensus diagnostic criteria by Graus et al. (4). More than half of the patients had in-hospital complications with a mortality rate of 19.4%. Treatment response varied according to age cohort, initial treatment scheme, and the time of treatment initiation. Although a progressive improvement in neurological disability was observed across all follow-up periods up to 1 year, no significant differences were identified between pediatric and adult patients in terms of clinical outcomes.

### Demographic and age-related symptoms

We observed a slight predominance of pediatric patients (57.6%), aligning with other cohort of anti-NMDA receptor AE from India (62% pediatrics) (9), and a male predominance in pediatrics versus a female predominance in adults, as previously reported by Zhao et al., in a systematic review of anti-NMDA receptor AE (10). The most common comorbidities in our study, such as COVID-19, obesity, and preexisting autoimmune disease, highlight the previously described association of AE with autoimmune, paraneoplastic or infectious triggers (11, 12). The shorter interval to admission (median of 7 days) in pediatric population, mostly associated with viral prodrome (13, 14) or more abrupt symptom onset, may reflect heightened caregiver vigilance, while adults may experience diagnostic delays due to nonspecific prodromal presentations.

The clinical presentation of AE remains heterogeneous, reflecting the involvement of multiple central nervous system regions. In our cohort, common initial symptoms included seizures, behavioral disorders, cognitive impairment, altered level of consciousness, and movement disorders, consisted with previous reports in diverse populations (15, 16). However, when comparing across age subgroups, we observed a more prominent presentation of acute neuropsychiatric symptoms in pediatric age group, including fever, altered consciousness, akinetic mutism, and motor and sensory deficits, than in adults (17). These findings suggest that pediatric AE are more likely to present multifocal neuropsychiatric symptoms rather than isolated clinical syndromes (18), likely due to ongoing neurodevelopment, receptor density, and myelination during normal development.

In contrast, adult patients more frequently exhibited a broader range of comorbidities at presentation. These discrepancies might also reflect differences in immune system maturity, blood–brain barrier permeability, and the frequency of paraneoplastic etiologies in adults. Moreover, the high prevalence of viral prodromes preceding neurological symptoms in pediatrics, combined with a greater frequency of systemic signs like fever, supports the hypothesis that post-infectious immune dysregulation plays a more prominent role in the pediatric subgroup (13, 14, 19). These findings reinforce the importance of age-adapted diagnostic approaches and suggest that future studies should investigate whether symptom clustering can help predict antibody status, disease severity, treatment modality and long-term outcomes in different age groups.

### Antibodies and imaging findings

Anti-NMDA receptor antibody-mediated AE was the most common etiology identified in our cohort, consistent with previous studies (20). Most of the patients (61.2%) had neuronal antibody testing. Limited access to antibody assays, often absent in public hospitals, contributes to underdiagnosis and delayed treatment in Latin America (6). This highlights the importance of availability of diagnostic tests for autoimmune encephalitis considering that this condition is treatable and delay in diagnosis or management can cause either severe disability or even death.

During an assessment of a suspected AE case, clinical history, neuroimaging (CT and MRI), EEG, and the search for mimicking etiologies are essential (21). We found that over 75% of the cases underwent these tests. Interestingly, nearly one-third of pediatric scans were normal, illustrating the clinical–radiologic paradox in pediatric AE (22). In contrast, adults showed more frequent MRI abnormalities, even though previous reports often showed normal imaging (23, 24). Conversely, EEG abnormalities—including diffuse temporal slowing, focal epileptiform discharges, and extreme delta brush in case of anti-NMDA receptor AE (25–27) were common across both groups, reinforcing the utility of EEG for early diagnosis, especially when antibody testing is delayed or unavailable.

### Immunotherapy and treatment response

The foundation of AE treatment lies in early immunotherapy which is associated with better clinical outcomes (28–30). However, around 30% of our cohort received antibiotics and antivirals, likely because infectious meningoencephalitis was an initial differential diagnosis in the emergency department. Otherwise, 92% of patients received first-line therapies, amongst which triple therapy was administered in 13.2% of cases although literature on this last approach is limited. Most of our patients showed a favorable response after treatment, consistent with a meta-analysis result, especially within 30 days since symptom onset (30). According to this, we observed that early treatment initiation (<7 days) correlated with sustained benefit in pediatric patients but not in adults, suggesting age-related differences in immunopathogenesis and neuroplasticity.

Although there is interest in comparing effectiveness between immunotherapies, we observe heterogeneity in treatment response. Our cohort showed similar outcomes for IVIG and plasma exchange monotherapy. However, some studies suggest plasma exchange may provide quicker relief by removing autoantibodies and inflammatory substances from plasma. Currently, there is no definitive evidence supporting the superiority of IVIG over plasma exchange (31).

### Complications, relapse, and follow-up

In-hospital neurological complications and infections occurred in over half of the cases, paralleling a Mexican cohort (32), and are likely related to prolonged hospital stays, ICU admission, and delayed initiation of immunotherapy (23). The observed in-hospital mortality rate of 9.7% was lower than a Chinese cohort, where it was associated with age over 45 years and antibody type (33). This difference may be related to higher proportion of pediatric patients in our sample and earlier initiation of immunotherapy. Variability in ICU access and local practices may also influence mortality outcomes across regions and antibodies (34).

Long-term neurological sequelae remain a major concern in autoimmune encephalitis. In our cohort, 32% of our patients experienced status epilepticus, and one-third required continued use of antiseizure medication beyond 6 months. These findings are consistent with previous studies reporting seizure recurrence rates of 39.6% (35), and epilepsy development in 28.4% of patients, particularly in those with super-refractory status epilepticus, abnormal EEG and MRI findings, antibody negativity, and delayed immunotherapy initiation (36, 37). At 12-month follow-up, 57.6% of our patients reported minor cognitive impairment, most frequently affecting memory and executive functions, as similar observed in an Argentinian study (38) and adults and pediatric cohorts (39, 40), where cognitive issues were more prevalent than behavioral symptoms.

Despite these sequelae, most patients in our study achieved functional independence (mRS < 2) at 6 months, consistent with international findings in both antibody-positive and seronegative populations (40, 41). However, according to a cohort from Mexico with anti-NMDA receptor AE, those patients with severe disability showed no improvement at follow-up (42). Prognosis in AE remains difficult to establish due to subtype variability, comorbidities, and limited long-term data. A key strength of our study is its multicentric design, which includes both pediatric and adult populations across multiple Latin American countries.

### Limitations and strengths

Given the rarity of the disease and the absence of standardized management protocols, the sample size for each treatment scheme was very heterogenous across pediatric and adult cohorts. So, we face challenges in conducting more in-depth statistical analyses of prognostic factors and individual treatment responses because they will be highly biased, limiting their interpretation. Additionally, the retrospective nature of the study resulted in some missing data, potentially limiting our conclusions. However, to our knowledge, this is the first regional multicenter study addressing clinical presentation, as well as follow-up and hospital outcomes in this disease in the region.

Previous multisite collaboration in Latin America in stroke (43), dementia (44), and multiple sclerosis (45) have successfully established research networks that support epidemiological surveillance, policy development, and the implementation of context-sensitive intervention. Building on these models, future efforts should focus on evaluating the cost-effective of local developed antibody testing assays and expanding telemedicine–based diagnostic networks to reduce delays in diagnosis and treatment. Furthermore, randomized controlled trials are essential to determine the optimal sequencing and duration of first- and second-line immunotherapies in resource-limited settings.

## Conclusion

This study represents the first regional multicenter analysis of the clinical presentation and outcomes of AE in Latin American hospitals, with balanced presentation of both pediatric and adult populations. In-hospital complications occurred in over half of the cases, with a mortality rate of 19.4%. Treatment response varied according to age cohort, initial treatment scheme, and time of treatment. Nearly half of the patients required ongoing psychotropic and antiseizure medications, and minor neurocognitive impairment emerged as the most frequent long-term sequelae. This study presents the initial results of the retrospective registry of the REAL LABIC project, representing a first step toward future prospective studies and subanalyses of patients with autoimmune encephalitis, where risk factors for clinical outcomes of interest in this patient population in Latin America can be better assessed.

## Data Availability

The raw data supporting the conclusions of this article will be made available by the authors, without undue reservation.

## References

[1] Patel A Meng Y Najjar A Lado F Najjar S. Autoimmune encephalitis: a physician’s guide to the clinical spectrum diagnosis and management. Brain Sci. (2022) 12:1130. doi: 10.3390/brainsci12091130, PMID: 36138865 PMC9497072

[2] Dalmau J Tüzün E Wu H y Masjuan J Rossi JE Voloschin A. Paraneoplastic anti–N-methyl-D-aspartate receptor encephalitis associated with ovarian teratoma. Ann Neurol. (2007) 61:25–36. doi: 10.1002/ana.21050, PMID: 17262855 PMC2430743

[3] Abboud H Probasco JC Irani S Ances B Benavides DR Bradshaw M. Autoimmune encephalitis: proposed best practice recommendations for diagnosis and acute management. J Neurol Neurosurg Psychiatry. (2021) 92:757–68. doi: 10.1136/jnnp-2020-325300, PMID: 33649022 PMC8223680

[4] Graus F Titulaer MJ Balu R Benseler S Bien CG Cellucci T. A clinical approach to diagnosis of autoimmune encephalitis. Lancet Neurol. (2016) 15:391–404. doi: 10.1016/S1474-4422(15)00401-9, PMID: 26906964 PMC5066574

[5] Dubey D Pittock SJ Kelly CR McKeon A Lopez-Chiriboga AS Lennon VA. Autoimmune encephalitis epidemiology and a comparison to infectious encephalitis. Ann Neurol. (2018) 83:166–77. doi: 10.1002/ana.25131, PMID: 29293273 PMC6011827

[6] Vasconcelos G d A Barreira RM Antoniollo KENT Pinheiro AMN Maia CFR Alves DMBS. Autoimmune encephalitis in Latin America: a critical review. Front Neurol. (2021) 11:606350. doi: 10.3389/fneur.2020.606350, PMID: 33551968 PMC7859257

[7] Vences MA Rivillas JA Campos-Gamarra RN Failoc-Rojas VE Godoy DA. Challenges in access to diagnosis and treatment of autoimmune encephalitis in hospitals in Latin America and the Caribbean. Acta Neurol Scand. (2025) 2025:1934971. doi: 10.1155/ane/1934971

[8] Vences MA Godoy DA Rivillas JA Reyes-Niño S García-Arellano M Melgarejo D. Registro de Encefalitis Autoinmune en países de Latinoamérica: proyecto REAL LABIC. Rev Neuropsiquiatr. (2025) 88:186–9. doi: 10.20453/rnp.v88i2.5512

[9] Raja P Shamick B Nitish LK Holla VV Pal PK Mahadevan A. Clinical characteristics, treatment and long-term prognosis in patients with anti-NMDAR encephalitis. Neurol Sci. (2021) 42:4683–96. doi: 10.1007/s10072-021-05174-6, PMID: 33728548

[10] Zhao X Teng Y Ni J Li T Shi J Wei M. Systematic review: clinical characteristics of anti-N-methyl-D-aspartate receptor encephalitis. Front Hum Neurosci. (2023) 17:1261638. doi: 10.3389/fnhum.2023.1261638 38053649 PMC10694196

[11] Sawalha A Alkilani H Abdelaziz R. The association between autoimmune encephalitis mediated by N-methyl-D-aspartate receptor autoantibodies and COVID-19: a systematic review. Encephalitis. (2024) 4:3–10. doi: 10.47936/encephalitis.2023.00171, PMID: 38126079 PMC11007402

[12] Lee KW Khan AHKY Ching SM Kumar SJ Raj CLVP Chia PK. Prevalence and factor associated with anti-N-methyl-D-aspartate receptor encephalitis among patients with medical conditions: a systematic review and meta-analysis. Neurol India. (2024) 72:476–86. doi: 10.4103/neuroindia.NI_981_20, PMID: 39041960

[13] Pruetarat N Netbaramee W Pattharathitikul S Veeravigrom M. Clinical manifestations, treatment outcomes, and prognostic factors of pediatric anti-NMDAR encephalitis in tertiary care hospitals: a multicenter retrospective/prospective cohort study. Brain Dev. (2019) 41:436–42. doi: 10.1016/j.braindev.2018.12.009, PMID: 30639077

[14] Wang W Li JM Hu FY Wang R Hong Z He L. Anti-NMDA receptor encephalitis: clinical characteristics, predictors of outcome and the knowledge gap in Southwest China. Eur J Neurol. (2016) 23:621–9. doi: 10.1111/ene.12911, PMID: 26563553

[15] Gong X Chen C Liu X Lin J Li A Guo K. Long-term functional outcomes and relapse of anti-NMDA receptor encephalitis: a cohort study in Western China. Neurol Neuroimmunol Neuroinflamm. (2021) 8:e958. doi: 10.1212/NXI.0000000000000958, PMID: 33589542 PMC8105891

[16] Zhang L Wu MQ Hao ZL Chiang SM Shuang K Lin MT. Clinical characteristics, treatments, and outcomes of patients with anti-N-methyl-D-aspartate receptor encephalitis: a systematic review of reported cases. Epilepsy Behav. (2017) 68:57–65. doi: 10.1016/j.yebeh.2016.12.019, PMID: 28109991

[17] Hardy D. Autoimmune encephalitis in children. Pediatr Neurol. (2022) 132:56–66. doi: 10.1016/j.pediatrneurol.2022.05.004, PMID: 35640473

[18] Mishra N Rodan LH Nita DA Gresa-Arribas N Kobayashi J Benseler SM. Encefalitis límbica asociada a anticuerpos anti-ácido glutámico descarboxilasa en un niño: ampliando el espectro de enfermedades cerebrales inflamatorias pediátricas. J Child Neurol. (2014) 29:677–83. doi: 10.1177/0883073813500527 24092895

[19] Abu Melha AA Aldress AS Alamri F. Prognostic factors and treatment outcomes in pediatric autoimmune encephalitis: a multicenter study. Front Neurol. (2024) 15:1441033. doi: 10.3389/fneur.2024.1441033, PMID: 39286808 PMC11402692

[20] Hiesgen J Schutte C. Autoimmune encephalitis: part 1 (epidemiology, pathophysiology and clinical spectrum). S Afr Med J. (2023) 113:116–21. doi: 10.7196/SAMJ.2023.v113i3.780, PMID: 36876355

[21] Dalmau J Graus F. Diagnostic criteria for autoimmune encephalitis: utility and pitfalls for antibody-negative disease. Lancet Neurol. (2023) 22:529–40. doi: 10.1016/S1474-4422(23)00083-2, PMID: 37210100

[22] Hacohen Y. Pediatric autoimmune neurologic disorders. Continuum (Minneap Minn). (2024) 30:1160–88. doi: 10.1212/CON.0000000000001464, PMID: 39088292

[23] Titulaer MJ McCracken L Gabilondo I Armangué T Glaser C Iizuka T. Treatment and prognostic factors for long-term outcome in patients with anti-NMDA receptor encephalitis: an observational cohort study. Lancet Neurol. (2013) 12:157–65. doi: 10.1016/S1474-4422(12)70310-1, PMID: 23290630 PMC3563251

[24] Dalmau J Gleichman AJ Hughes EG Rossi JE Peng X Lai M. Anti-NMDA-receptor encephalitis: case series and analysis of the effects of antibodies. Lancet Neurol. (2008) 7:1091–8. doi: 10.1016/S1474-4422(08)70224-2, PMID: 18851928 PMC2607118

[25] Schmitt SE Pargeon K Frechette ES Hirsch LJ Dalmau J Friedman D. Extreme delta brush: a unique EEG pattern in adults with anti-NMDA receptor encephalitis. Neurology. (2012) 79:1094–100. doi: 10.1212/WNL.0b013e3182698cd8, PMID: 22933737 PMC3525298

[26] Sonderen AV Arends S Tavy DLJ Bastiaansen AEM Bruijn MAAM Schreurs MWJ. Predictive value of electroencephalography in anti-NMDA receptor encephalitis. J Neurol Neurosurg Psychiatry. (2018) 89:1101–6. doi: 10.1136/jnnp-2018-318376, PMID: 30135187

[27] Jeannin-Mayer S André-Obadia N Rosenberg S Boutet C Honnorat J Antoine JC. EEG analysis in anti-NMDA receptor encephalitis: description of typical pat terns. Clin Neurophysiol. (2019) 130:289–96. doi: 10.1016/j.clinph.2018.10.017, PMID: 30611120

[28] Irani SR. Autoimmune Encephalitis. Continuum (Minneap Minn). (2024) 30:995–1020. doi: 10.1212/CON.0000000000001448, PMID: 39088286

[29] Thompson J Bi M Murchison AG Makuch M Bien CG Chu K. The importance of early immunotherapy in patients with faciobrachial dystonic seizures. Brain. (2018) 141:348–56. doi: 10.1093/brain/awx323, PMID: 29272336 PMC5837230

[30] Nosadini M Eyre M Molteni E Thomas T Irani SR Dalmau J. Use and safety of immunotherapeutic management of N-methyl-D-aspartate receptor antibody encephalitis: a meta-analysis. JAMA Neurol. (2021) 78:1333–44. doi: 10.1001/jamaneurol.2021.3188, PMID: 34542573 PMC8453367

[31] Zhang Y Liu G Jiang M Chen W Su Y. Eficacia del intercambio plasmático terapéutico en pacientes con encefalitis refractaria grave a fármacos anti-receptor NMDA. Neurotherapeutics. (2019) 16:828–37. doi: 10.1007/s13311-019-00725-4 30868469 PMC6694354

[32] Martínez-Angeles V Ramírez-Bermúdez J Espínola-Nadurille M López-Hernández JC Martínez-Carrillo FM Rivas-Alonso V. Complicaciones intrahospitalarias en pacientes con encefalitis anti-NMDAR definida en un centro de tercer nive. Arch Neurocien. (2024);29. Available online at: https://archivosdeneurociencias.org/index.php/ADN/article/view/563 (accessed April 12, 2025)

[33] Zhong R Chen Q Zhang X Zhang H Lin W. Risk factors for mortality in anti-NMDAR, anti-LGI1, and anti-GABABR encephalitis. Front Immunol. (2022) 13:845365. doi: 10.3389/fimmu.2022.845365, PMID: 35320933 PMC8934853

[34] Kvam KA Stahl JP Chow FC Soldatos A Tattevin P Sejvar J. Outcome and sequelae of autoimmune encephalitis. J Clin Neurol. (2024) 20:3–22. doi: 10.3988/jcn.2023.0242, PMID: 38179628 PMC10782092

[35] Chen S s Zhang Y f Di Q Shi J p Wang L l Lin X j. Predictors and prognoses of epilepsy after anti-neuronal antibody-positive autoimmune encephalitis. Seizure. (2021) 92:189–94. doi: 10.1016/j.seizure.2021.09.007, PMID: 34551365

[36] Zhong R Zhang X Chen Q Li M Guo X Lin W. Acute symptomatic seizures and risk of epilepsy in autoimmune encephalitis: a retrospective cohort study. Front Immunol. (2022) 13:813174. doi: 10.3389/fimmu.2022.813174, PMID: 35281052 PMC8904420

[37] Huang W Zhang H Li X Zhang J Chen J Chen Z. Prognostic factors underlying the development of drug-resistant epilepsy in patients with autoimmune encephalitis: a retrospective cohort study. J Neurol. (2024) 271:5046–54. doi: 10.1007/s00415-024-12432-y, PMID: 38801431

[38] Palacios S. Manin A. Dorman G. S. Kim L. M. Núñez M. R. Villa A. M. (2023). Secuelas cognitivas en encefalitis inmunomediadas: cohorte de pacientes en Argentina. Medicina, 83, 402–410. Available online at: https://www.medicinabuenosaires.com/revistas/vol83-23/n3/402.pdf 37379537

[39] Finke C Kopp UA Prüss H Dalmau J Wandinger KP Ploner CJ. Cognitive deficits following anti-NMDA receptor encephalitis. J Neurol Neurosurg Psychiatry. (2012) 83:195–8. doi: 10.1136/jnnp-2011-300411, PMID: 21933952 PMC3718487

[40] Gadian J Eyre M Konstantoulaki E Almoyan A Absoud M Garrood I. Neurological and cognitive outcomes after antibody-negative autoimmune encephalitis in children. Dev Med Child Neurol. (2022) 64:649–53. doi: 10.1111/dmcn.15101, PMID: 34724211

[41] Huang T Liu F Wang B Wang C Hao M Guo S. Clinical characteristics and prognosis in patients with neuronal surface antibody-mediated autoimmune encephalitis: a single-center cohort study in China. Front Immunol. (2023) 14:1213532. doi: 10.3389/fimmu.2023.1213532, PMID: 38152405 PMC10751914

[42] Herrera-Mora P Munive-Baez L Ruiz García M Galindo-Martínez A Maldonado-Diaz DE Delgado RD. Anti-N-methyl-D-aspartate receptor encephalitis: an observational and comparative study in Mexican children and adults. Clin Neurol Neurosurg. (2021) 210:106986. doi: 10.1016/j.clineuro.2021.106986, PMID: 34688092

[43] Muñoz-Venturelli P González F Urrutia F Mazzon E Navia V Brunser A. Stroke care and collaborative academic research in Latin America. Salud Publica Mex. (2022) 64:S40–5. doi: 10.21149/12803, PMID: 36130397

[44] Ibanez A Yokoyama JS Possin KL Matallana D Lopera F Nitrini R. The multi-partner consortium to expand dementia research in Latin America (ReDLat): driving multicentric research and implementation science. Front Neurol. (2021) 12:631722. doi: 10.3389/fneur.2021.631722/full 33776890 PMC7992978

[45] Rojas JI Carnero Contentti E Abad P Aguayo A Alonso R Bauer J. Research priorities in multiple sclerosis in Latin America: a multi-stakeholder call to action to improve patients care: research priorities in MS in LATAM. Mult Scler Relat Disord. (2021) 53:103038. doi: 10.1016/j.msard.2021.103038, PMID: 34090128

